# Self-reported knowledge and skills related to diagnosis and management of keratoconus among public sector optometrists in the Limpopo province, South Africa

**DOI:** 10.4102/phcfm.v14i1.3668

**Published:** 2022-12-15

**Authors:** Pheagane M.W. Nkoana, Vanessa R. Moodley, Khathutshelo P. Mashige

**Affiliations:** 1 Discipline of Optometry, Faculty of Health Sciences, University of KwaZulu-Natal, Durban, South Africa; 2 Department of Optometry, Faculty of Health Sciences, University of Limpopo, Polokwane, South Africa

**Keywords:** keratoconus, knowledge and skills of keratoconus, optometrist, Capricorn district of the Limpopo province, Contact lenses

## Abstract

**Background:**

Keratoconus (KC) has been regarded as a rare condition, although recent studies, including those in South Africa, suggest it is common and is increasing in prevalence. Furthermore, South African-based studies have shown that KC is normally detected at advanced or severe stages. Knowledge and skills for examination, diagnosis and management of KC by optometrists are important for the early detection and management of the disease.

**Aim:**

This study aimed to evaluate self-reported knowledge and skills for examination, diagnosis and management of KC patients among public sector optometrists.

**Setting:**

Seven public hospitals of the Capricorn district of the Limpopo province, South Africa.

**Methods:**

A quantitative cross-sectional descriptive study design was used. A self-administered online questionnaire was used to collect data on demographic characteristics of participants and their knowledge, skills and practice for the diagnosis and management of KC.

**Results:**

Twenty-four optometrists (*n* = 24) with a mean age of 39 ± 5.67 years, 18 (75%) of whom were female, participated in the study. Nineteen (79%) reported that their hospitals did not have the appropriate equipment to examine, diagnose and manage KC patients. Lack of equipment, poor knowledge, skills and competencies, hospital’s level of services, policy and lack of interest were cited as barriers to contact lens fittings in KC patients. Using a dichotomised summation of self-reported knowledge and skills of KC, 13 (54.2%) of the optometrists were knowledgeable and skilled on risk factors of KC and examining, diagnosing and managing KC patients.

**Conclusion:**

A significant proportion of optometrists did not have the appropriate knowledge and skills to examine, diagnose and manage KC patients. Lack of equipment and poor knowledge and skills were the main barriers to contact lens fittings in managing KC.

**Contribution:**

This article highlights the need for the district to upskill the optometrists through a structured programme with a theory and practical component and also provide the necessary equipment to enhance patient care.

## Introduction

Keratoconus (KC) is a progressive, noninflammatory, bilateral corneal ectasia that presents with thinning and protrusion, resulting in high myopia and irregular astigmatism.^1,2^ Although it may be observed earlier or later, its onset is usually around puberty^3,4^ and stabilises in the fourth decade of life.^2,5,6^ A recent review by Hashemi et al.^1^ estimated a global prevalence of 1.38 per 1000 persons from a sample of 50 358 341 across 29 countries.

The early stages of KC are characterised by blurred or distorted vision, constant eye rubbing, Charleaux’s oil droplet sign, scissors reflex with retinoscopy and frequent changes of spectacles, although better visual acuity may still be achieved with spectacles.^2,7^ As the condition progresses, more marked corneal changes appear, such as Fleischer’s ring, Vogt’s striae, increased corneal nerve visibility and scarring.^2,8^ Munson’s sign, Rizutti’s sign, corneal hydrops, stromal oedema, and stromal scarring are common characteristics of severe KC.^8,9^

The classification and management of KC are dependent on the clinical presentation, which becomes more pronounced as the condition progresses.^7^ Nascent KC can be managed with spectacles and soft contact lenses, but rigid and scleral lenses are indicated for more severe stages of KC.^8^ Corneal cross-linking (CXL) may be performed at early stages of KC in rapid progressive cases and keratoplasty in severe stages of KC when other methods cannot improve vision to satisfactory levels.^4,9^

Current management protocols of KC promote early detection to manage the symptoms, provide vision correction, arrest progression and rehabilitate visual loss using spectacles, contact lenses or surgical procedures as indicated.^2,10,11,12,13^ Traditional methods of diagnosing KC such as ascertaining clinical symptoms and risk factors through a case history and performing a slit lamp assessment, keratometry and retinoscopy^7,14,15^ are still effective. This equipment is the prescribed minimum by the Health Professions Council of South Africa (HPCSA).^16^ Newer technologies such as pachymetry, ocular coherence tomography and corneal topography^12^ have enhanced the ability of eye care practitioners to diagnose and manage the condition.

There has not been a national screening programme in South Africa; hence, prevalence or KC and the rate of undiagnosed patients has not been reported yet. However, anecdotal evidence suggests that KC is prevalent in patients presenting at public hospitals in the Capricorn district of the Limpopo province, South Africa. The service model used in these public facilities is such that optometrists provide primary eye care services at all levels of hospitals and, additionally, support community health centres and clinics.^17^ The scope of practice in South Africa defines optometrists as primary eye care practitioners who diagnose and manage visual errors with ocular devices, including spectacles and contact lenses, for conditions such as KC.^18^ In the Limpopo province, trained healthcare providers such as ophthalmic nurses are only available at district and tertiary-level hospitals, and ophthalmologists are only available at tertiary-level facilities. This lack impacts the ability to diagnose and treat KC. They are responsible for managing conditions associated with KC and also perform surgical interventions when indicated by ophthalmologists.

Early detection and appropriate management of KC patients require optometrists to be knowledgeable and clinically competent and have appropriate infrastructure. A lack of these will potentially contribute to misdiagnoses and underdiagnoses,^19,20,21,22^ with resultant poor prognoses and diminished quality of life of the affected patients. Considering the observed increase in KC cases in these facilities, this study was conducted to establish whether optometrists, as primary eye care practitioners, were sufficiently knowledgeable and skilled in the diagnosis and management of KC.

## Research methods and design

### Study design

This quantitative, cross-sectional, descriptive study design was conducted over 3 months, commencing in July 2020. Self-administered online questionnaires were used with the aim of evaluating the self-reported knowledge and skills of diagnosing and managing KC. The study design allowed researchers to reach optometrists who were inaccessible because of geographical location.

### Setting

The study was conducted in public hospitals within the Capricorn district, Limpopo province, South Africa. The Capricorn district has eight public hospitals, seven of which consented to participate in the study. Six of the hospitals, Botlokwa, Helen Franz, Lebowakgomo, Seshego, W.F. Knobel and Zebediela are district hospitals, and Mankweng is a tertiary hospital. Mankweng, Lebowakgomo and Seshego are located in townships or semi-urban areas, although they, like the other hospitals, were mostly serving persons from rural areas. The hospitals employ optometrists and provide comprehensive eye care service to the general public within the district. Service includes screening, examination, diagnosis and management of eye conditions in a controlled hospital setting, as well as outreach programmes.

### Study population

All 24 optometrists employed in the seven public hospitals of the Capricorn district were included in the study. Purposive sampling was used to select the optometrists because of their common shared characteristics. After gatekeeper permissions were granted by hospitals, optometrists were invited to participate in the study through convenient online media platforms including e-mail and WhatsApp. They all gave consent of participation.

### Data collection

A self-administered online questionnaire was developed, piloted and used to collect data in the study.

#### Development of the questionnaire

The questionnaire, consisting of closed-ended questions on practitioner demography, work experience, KC knowledge and skills, equipment availability and current KC-related diagnosis and management practices, was developed from existing literature and practice. This study also explored possible barriers experienced by optometrists to KC diagnosis and management. The item questions that assessed the level of knowledge and skills were measured on a five-point Likert scale ranging from poor = 1, fair = 2, average = 3, good = 4 to excellent = 5. The total summation of all six items was calculated, and optometrists whose total summation was equal to or above the median score (50% more) were considered knowledgeable.

Google Forms was used to administer the questionnaires, which were completed and returned online. Data from these questionnaires were captured onto a Microsoft Excel spreadsheet.

#### Validity of the questionnaire

The questionnaire used was developed by the primary author and reviewed by the secondary authors. It was then validated by a panel of eight experts in the field of optometry, some of whom were academicians and some of whom were in public and private practice. The Pearson product moment correlations test was conducted on all knowledge and skills items, which were found to be valid, and their count values were greater than the critical values at 0.05 significance. Hence, it was concluded that all the knowledge and skills questions were valid.

#### Reliability of the questionnaire

Test–retest reliability was conducted by re-administering the questionnaire to the first five (20.8%) respondents 10 days after their first encounter so as to check for consistency. For internal consistency, Cronbach’s alpha was computed for all knowledge and skills question results, which showed high correlation of items, and also a score of 0.79 was attained, indicating reliability of the questionnaire.

### Data analysis

Data were captured onto an Excel spreadsheet and exported to the Statistical Package for Social Sciences (SPSS) version 26 for analysis. Descriptive statistics, including frequencies and percentages, mean values, standard deviations and ranges, were calculated. Bivariate analysis was used to analyse the relationship between variables using a 5% significance level (*p* = 0.05).

### Ethical considerations

Ethical clearance to conduct the study was obtained from the Biomedical Research Ethics Committee of the University of KwaZulu-Natal (ref. no. BREC/000.01223/2020). Permission to conduct the study was also obtained from the Limpopo Province Department of Health (reference number LP-202005-002).

## Results

Table 1 shows the demographic characteristics of the participants. All 24 optometrists returned completed questionnaires; hence, the study attained a 100% response rate. Twenty-four optometrists with a mean age of 39 ± 5.67 years participated in the study. Eighteen (75.0%) were female and six (25.0%) were male. All optometrists were black Africans. The majority of practitioners (87.5%) had more than 5 years of working experience in public service, and 66.7% had occupied their current post for more than 5 years. Four (16.7%) optometrists had changed their place of work in the past 5 years or less and only one (4.2%) in the past 11–15 years.

**TABLE 1:** Demographic characteristics of the participants (*n* = 24).

Variable	*n*	%	Mean ± s.d
**Gender**			
Male	6	25.0	-
Female	18	75.0	-
**Age**	-	-	39 ± 5.67
**Years spent in public service**			
≤ 5	3	12.0	-
6–10	9	37.5	-
11–15	6	25.0	-
> 15	6	25.0	-
**Years in the current post**			
≤ 5	8	33.3	-
6–10	8	33.3	-
11–15	6	25.0	-
> 15	2	8.4	-
**Type of hospital**			
District	8	33.3	-
Tertiary	16	66.7	-

**FIGURE 1:**
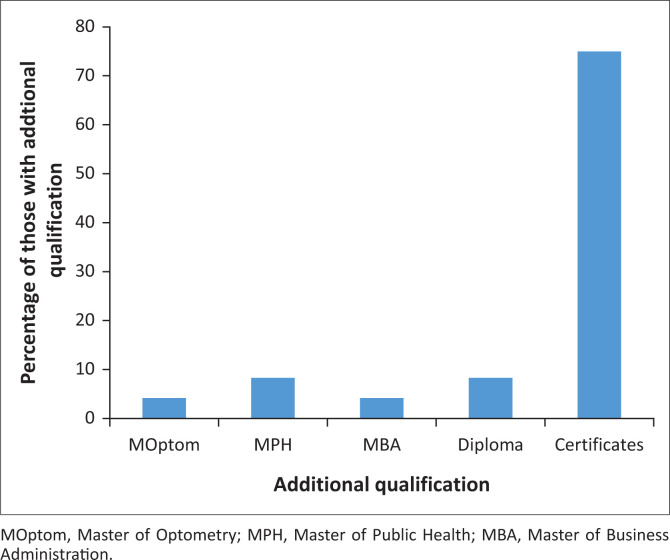
Highest additional qualifications attained.

Figure 1 shows additional qualifications attained. Four (16.7%) optometrists had acquired master’s qualifications and others had either a diploma or certificate.

Table 2 shows the number of equipment available at each of the seven hospitals. Mankweng and Lebowakgomo hospitals had two refraction units (of ophthalmic chair and stand, projector, phoropter and/or trial case and frame) each, while other hospitals had one refraction unit each. Regarding retinoscope and ophthalmoscopes, Mankweng hospital had four units, Botlokwa and Helen Franz had two units each and all other hospitals had one unit each. Three (42.8%) hospitals did not have a slit lamp biomicroscope, and four (57.1%) did not have a keratometer. Mankweng hospital reported waiting for delivery of a Pentacam during the time of the study. None of the other hospitals had other equipment such as pachymeters and topographers. No other relevant equipment for KC examination was available.

**TABLE 2:** Equipment available in each hospital.

Equipment	Bot	HFr	Leb	Man	Ses	WFK	Zeb
Refraction unit	1	1	2	2	1	1	1
Retinoscopy and ophthalmoscopy	2	2	1	4	1	1	1
Slit lamp	0	1	0	2	1	0	1
Keratometer	0	0	0	2	1	0	1

Bot, Botlokwa; HFr, Helen Franz; Leb, Lebowakgomo; Man, Mankweng; Ses, Seshego; WFK, W.F. Knobel; Zeb, Zebediela.

After dichotomising the summation of the total score of the six items into knowledgeable (score equal to or above the median of 50%) or not knowledgeable, 13 (54.2%) optometrists were found to be knowledgeable about KC, and 11 (45.8%) were not knowledgeable.

Table 3 shows the practitioners’ self-reported perceptions of their knowledge and skills regarding diagnosis and management of KC. A total of 15 (62.5%) optometrists reported having good knowledge and skills of the risk factors for KC; 10 (41.7%) had good knowledge and skills of screening, examination and diagnosis of KC; 9 (37.5%) had good knowledge and skills of ocular pathological conditions; and 8 (33.3%) had good knowledge of the associated systemic conditions. The majority (58.3%) reported having poor knowledge of surgical interventions of KC.

**TABLE 3:** Knowledge and skills of keratoconus as self-reported by optometrists.

Item	Poor		Fair		Average		Good		Excellent	
	*n*	%	*n*	%	*n*	%	*n*	%	*n*	%
Risk factors	-	-	1	4.2	6	25.0	15	62.5	2	8.3
Screening, examination and diagnosis	1	4.2	-	-	8	33.3	10	41.7	5	20.8
Management with nonsurgical interventions	2	8.3	4	16.7	11	45.9	5	20.8	2	8.3
Management with surgical interventions	14	58.3	5	20.8	4	16.7	1	4.2	-	-
Associated systemic conditions	4	16.7	2	8.3	8	33.4†	8	33.3	2	8.3
Associated ocular pathological conditions	2	8.3	2	8.3	6	25.0	9	37.6†	5	20.8

† , Rounded off to the next 0.1.

Six (25.0%) optometrists indicated that they received training on examination, diagnosing and managing KC. Of the training in KC received by optometrists, 5 (20.8%) attended a presentation in a continuous professional development (CPD) workshop, and only one attended a workshop with a practical component that included fitting contact lenses on KC patients. Of the five optometrists who attended training, 2 (33.3%) were in the past year, 3 (50%) were in the past 3 years and 1 (17%) was in the past 5 years. However, all 24 (100%) optometrists, including those who had received training, indicated that they required some form of additional training and would like to attend training.

**FIGURE 2:**
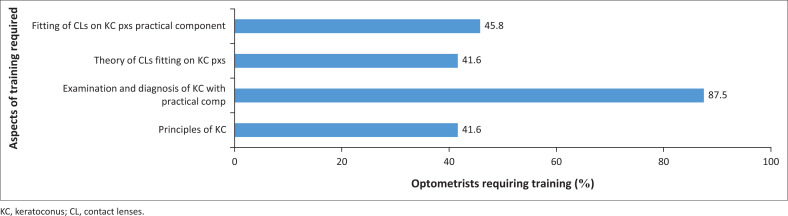
Type of training required by optometrists.

Figure 2 shows the aspects of training required by optometrists. The majority (87.5%) of optometrists reported that they require training in theory and practice related to examination and diagnosis of KC patients, while a further 45.8% reported requiring training in theory and practice of fitting contact lenses on KC patients. A total of 10 (41.6%) optometrists required training in the principles of KC, and another 10 (41.6%) required training in the fitting of contact lenses on KC patients.

Table 4 shows the demographic variables associated with the knowledge and skills of optometrists regarding KC. There was no significant difference in findings between male and female optometrists (*p* = 0.125); those in district and tertiary hospital types (*p* = 0.285); whether optometrists were initially trained in managing KC or not; or by the number of years optometrists had in practice post their graduation (*p* = 0.161 to *p* = 0.702). In a bivariate analysis, male optometrists were six times more likely to be knowledgeable compared with female optometrists (odds ratio [OR] = 6.25, [95% CI: 0.60; 64.86]). Optometrists in the tertiary Mankweng hospital were three times more likely to be knowledgeable than those in district hospitals (OR = 2.81, [95% CI: 0.42; 18.73]). There was no likelihood that optometrists who attended some form of additional postgraduate or CPD training had reported better knowledge and skills about KC than those who did not. Instead, the optometrists who never attended any training had three times more likelihood of superior self-reported knowledge and skills about KC (OR = 3.14, [95% CI: 0.45; 21.96]).

**TABLE 4:** Demographic factors associated with keratoconus knowledge and skills.

Variable	OR	95% CI	*p*
**Gender**			
Female	Ref.	-	-
Male	6.25	0.60; 64.86	0.125
**Hospital type**			
District	Ref.	-	-
Tertiary	2.81	0.42; 18.73	0.285
**Years in public service**			
≤ 5	Ref.	-	-
6–10	1.75	0.09; 30.83	0.702
11–15	0.50	0.03; 8.95	0.638
> 15	0.10	0.03; 2.05	0.161
**Trained managing KC**			
Yes	Ref.	-	-
No	3.14	0.45; 21.96	0.248

KC, keratoconus; OR, odds ratio; CI, confidence interval.

**FIGURE 3:**
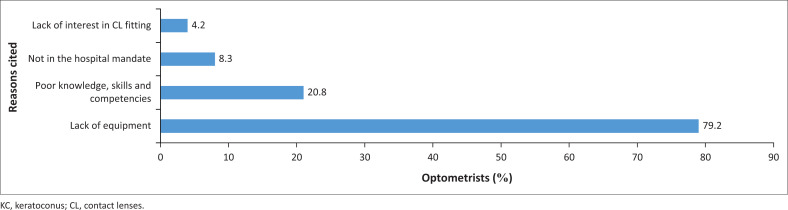
Reasons cited for not effectively diagnosing and managing keratoconus with contact lenses.

Figure 3 shows common reasons cited by optometrists as barriers to effective diagnosis and management of KC with contact lenses. Nineteen (79.2%) optometrists cited lack of appropriate equipment and 5 (20.8%) reported lack of knowledge, skills and competencies. Of the 5 (20.8%) who had appropriate equipment at their hospital, only three (12.5%) fitted contact lenses, 1 (4.0%) did not fit contact lenses, citing a lack of knowledge, skills and competencies, while the other (4.0%) indicated a lack of interest in contact lenses fitting.

## Discussion

This study aimed to assess the level of self-reported knowledge and skills in the examination, diagnosis and management of KC by optometrists in the public hospitals of Capricorn district, Limpopo province, South Africa. Optometrists’ knowledge and skills in the diagnostic and referral patterns of KC patients optimise patient care^19^ as they play a critical role in the diagnosis and management of KC. However, little is known about their knowledge and skills regarding the diagnosis and management of KC and other chronic eye diseases.^19,20^ In an eye care service-level setup as in South Africa, where most patients with KC are likely to be under the care of optometrists, their knowledge and skills of the condition are important for patient care optimisation, consideration of co-management opportunities and future training.^19^

The study revealed that mostly young adults, with a mean age of 39 years (s.d. ± 5.67), were employed as optometrists in the study facilities, which may be attributed to the fact that the Department of Health in this province started with the training and employment of optometrists only in the past two decades. Research indicates^23,24^ that the benefits of having a younger staff complement are that they are more likely to be flexible and adaptable to working environments, resilient, optimistic and engage in skill development through research and information evaluation. They also have better physical and mental health, engage in self-improvement through learning and academic achievements, are more positive and seem to enjoy their lives better than older professionals. They are therefore more likely to be able to engage in self-development, especially through learning, when required. Serving 84% of the population in South Africa,^25^ public sector facilities generally have very high daily patient rates and long working hours, demanding good physical health and youthful energy levels in health workers, which may make this study cohort physically fit for purpose. While it is beneficial to employ younger staff members, a good balance between the younger and older staff members should be maintained.^26^ The latter usually complement some of the attributes lacking in a younger staff complement.^26^ They are usually more experienced and skilled and have better consistency and direction, hence providing stability in an organisation, and they further have a better compliance and adherence to organisational boundaries.^26^

Similar to the findings of Gcabashe et al.^27^ in their public sector study in the KwaZulu-Natal province, there were predominantly more female (75%) than male (25%) optometrists. The difference in the proportion of women to men in both these studies may be attributed to the predominance of women in the registration statistics of the HPCSA and student intake in South African universities offering optometry programmes.^27^ This phenomenon has also been observed as a growing global trend, as shown in the United States of America where almost 70% of students in most classes are women.^28,29,30^ The predominance of women in public sector employment may be because of the job security, better work–life balance within the public sector and reluctance to take risks in the current over-burdened global economy.

Most optometrists (87.5%) had many years of experience serving the public sector, and the 100% retention rate with low staff turnover augurs well for the public health sector in the Limpopo province. This finding is contrary to the study by Buthelezi and Van Staden,^31^ which found that at least 45% of the KwaZulu-Natal public service optometrists indicated an intention to leave their employment. A review of the reasons contributing to the success of retention rates in the Capricorn district of Limpopo could be investigated by those provinces struggling to retain optometrists in their employ. Having the majority of optometrists being experienced practitioners will be expected to contribute positively to clinical eye care services at their respective facilities. There is, however, a general challenge with retention rates of health workers within the public sector, with staff often leaving or intending to leave.^30,31,32,33^

The availability of equipment was evaluated to determine the facility infrastructure’s suitability for fitting contact lenses or conducting follow-up consultation visits for contact lens–wearing patients. Despite all seven hospitals having refraction units, retinoscopes and ophthalmoscopes, of concern is that only three hospitals had keratometers and slit lamp biomicroscopes. The lack of critical equipment indicates that the facilities do not meet the minimum equipment requirements for contact lens fitting as stipulated by the HPCSA, the regulatory authority.^16^ Slit lamp and keratometer or topographer are part of the basic equipment requirements for examination, diagnosis, grading of KC, fitting of contact lenses and post-fitting management of contact lens complications.^8,34,35^ Furthermore, patients at risk of KC are usually preteens and teenagers and require timely diagnosis and management with corrective devices to minimise long-term visual morbidity and, where indicated, CXL to retard progression.^36,37^ The lack of basic equipment for the early diagnosis will compromise patient care in general and potentially have negative long-term quality of life consequences. Corneal cross-linking retards KC progression but needs a threshold corneal thickness of 400 µm to be performed. Therefore, late detection and diagnosis of KC deprives children of the opportunity to retard the progression of the condition if the corneal thickness reduces to below the eligibility threshold for CXL.^38^ The long-term economic and visual morbidity impact warrants a public health intervention.

A dichotomised summation of self-reported clinical knowledge and skills about KC of optometrists revealed that 46% of optometrists in these hospitals lacked the basic knowledge and skills for KC patient care. Despite all having over 5 years’ experience in optometry practice, their knowledge and skills for examining, diagnosing and managing KC were not adequate. With limited knowledge and skills, there is a high likelihood of underdiagnosing, misdiagnosing and poor management of diseases.^39^ The consequence of the lack of adequate skills and knowledge is that KC may only be diagnosed at later stages, when signs and symptoms are more advanced and apparent, and the quality of the patient’s life is significantly diminished.

To the best of our knowledge, no similar study investigating the knowledge and skills of KC was conducted with optometrists, and only one such study was conducted with ophthalmologists at a tertiary referral centre in Lucerne, Switzerland.^20^ The study found that none of the ophthalmologists had minimal keratoconus knowledge (MKK) concerning its definition, risk factors, symptoms and possible treatment options. However, they had a mean MKK of 52%, with a range between 28.6% and 81.0%. Lacking MKK among ophthalmologists implied that there was a possibility that the KC was poorly detected, diagnosed and reported on in Switzerland as compared with other countries. Similar to the findings of Baenninger et al.,^20^ KC may be poorly detected, diagnosed, managed and reported on in the Capricorn district because of the lack of basic equipment and lack of adequate knowledge and skills of optometrists in KC.

Upon evaluation of the various elements of the MKK separately, almost all optometrists (96%) had cumulatively rated their knowledge and skills of risk factors, screening, examination and diagnosis of KC range from average to excellent. This is commendable in patient care. In a resource-constrained environment such as public hospitals in South Africa, where there is a lack of equipment, this will enable optometrists to develop a protocol for optimising KC patient risk assessment, screening, examination and diagnosis.^40,41^ They can develop protocols for early detection of KC in line with possible available equipment and resources for patient care.^37^

The majority of the optometrists (75.0%) rated their knowledge of nonsurgical interventions from average to excellent. It is, however, concerning that a significant proportion (25.0%) reported poor to fair knowledge of surgical interventions of KC. These findings imply that patients are not being provided with the most appropriate management intervention strategy. Sound knowledge of intervention strategies, both surgical and nonsurgical, enhances clinical decision-making and supports creativity in patient management.^42^ For best practice, optometrists need to develop protocols for patient management, which may be based on the degree of progression of the condition. The protocol may specify the grading of KC, referral guidelines and devices used for each grade.

At least 25.0% of optometrists reported having attended presentations as part of CPD workshops; however, all reported that they needed further training with an emphasis on practical components. Furthermore, they expressed the need for training on general principles of KC, theoretical and practical components in examining, diagnosing and managing KC patients and the theory and practice of fitting contact lenses for management of KC patients. This may indicate that attending CPD workshops did not have significant impact or that the knowledge and skills gap was so significant that it could not be adequately closed by the regular CPD activities offered. Continuous professional development activities are beneficial in providing appropriate knowledge and skills to the professionals using formal or structured methods, although there is limited knowledge on how they impact patient outcomes.^43^ Continuous professional development activities are usually mandatory in many countries, including South Africa. Their quality plays a significant role in the development process of professionals, although in some instances, professionals attend to meet the requirements of the legislative bodies, with less consideration of the likely impact or outcomes.^43,44^

The optometrists in this study may need a structured, formal training programme which is periodical and which has both theoretical and practical components to help bridge the knowledge and skills gap among these optometrists. In addition, informal, continuous and volitional peer support groups, mentorship programmes, observations and team meetings or briefings^44^ facilitated by those optometrists who manage KC patients regularly may assist in improving KC patient care within and across the hospitals in the district. Although there is enhanced daily professional experience in the latter approach, its impact is usually limited by staff shortages and workloads.^43^

A bivariate analysis of the self-reported knowledge and skills of optometrists showed a higher likelihood that recent graduates who were male, employed at the tertiary hospital and did not undergo additional training were most likely to be more knowledgeable than their counterparts. These findings had higher *p*-values, suggesting poor significance, owing to limitations such as sample size. Male optometrists were generally newly qualified in comparison and hence may have better knowledge and skills retention from their more recent undergraduate training. The majority were woman (75%) who had been in employment and may either have not had the same level of undergraduate training or have retained less of the acquired knowledge and skills over the years because of KC management not being part of the clinical protocols at their respective hospitals. Contrary to this concession, the study by Hodge et al.^19^ found more experienced optometrists to be more knowledgeable, skilled and competent to examine, diagnose and manage KC. This study highlights a need for further investigation into reasons contributing to the self-reported lack of adequate KC knowledge and skills of optometrists, despite having many years of work experience.

Contact lenses were reported to be fitted only at Mankweng hospital, and only three of the five optometrists at the facility fit contact lenses. As this facility is a referral centre for all other hospitals, having only three optometrists for the entire district is inadequate, as these optometrists will have to do diagnosis, contact lens fittings and aftercare consultations. There is a likelihood that these patients had long waiting periods for their referring optometrists to secure appointments and hence present exhibiting late signs of KC. Patients may experience challenges including requiring more time to complete consultation at the tertiary hospital compared with their local district hospital, inconvenience with distance, transportation and travelling times and also the costs associated with travelling, which have potential to increase patient dropout.^45^ Ideally, all optometrists at district and tertiary-level hospitals should be competent to diagnose KC at an early stage and manage patients appropriately to improve vision and prevent the progression of the disease.

The majority of optometrists (79.2%) reported equipment as the main barrier to screening, examination, diagnosis, management of KC and contact lens fitting, which has also been a common finding in earlier^14,35^ and more recent studies^46,47^ across many fields. Practitioners agree that it has a negative consequential impact on KC patient care, as care is adjusted to accommodate available resources in a facility.^14,25^ In South Africa, a lack of equipment is not only limited to the Capricorn district; a similar situation was reported in the KwaZulu-Natal public hospitals, where hospitals could not offer some optometry services related to binocular vision care, paediatric optometry and contact lenses.^31^ Besides South Africa, many other low- and middle-income countries face similar challenges.^48^ Optometrists tend to cancel or reschedule their services to cope with breakdowns or wear and tear of equipment because of overuse in well-equipped hospitals.^48^ Hospitals need to acquire appropriate equipment that enables early detection of KC and effective fitting of contact lenses such as corneal topographers and ocular coherence tomographers to enhance patient management.

In cases where equipment was available, lack of knowledge and skills was also cited by 20.8% of optometrists as a barrier to screening, examining, diagnosing and managing KC patients, especially with contact lenses. Irrespective of previous training, some were still not knowledgeable, suggesting that the training may be ineffective. Optometrists may require structured programmes or regular reskilling workshops and other forms of knowledge enhancement such as case studies. Upskilling programmes are one effective approach to providing knowledge and skills to healthcare professionals.^49^

Other barriers reported were hospital policy, as optometrists (8.3%) indicated that contact lens fitting was not in the mandate of the hospitals. In addition, some optometrists (4.2%) reported less interest in contact lenses fitting. This is contrary to the principles of patient care, because this policy on contact lens fitting and dispensing in public hospitals conflicts with the regulated scope of practice of optometrists about fitting and dispensing contact lenses as outlined by the HPCSA.^18,50^ Practitioners affected by this policy mandate may be regarded as providing a compromised level of service because they do not provide contact lenses. The study by Buthelezi and Van Staden also reported contact lens fitting as a barrier to KC management in the public service of KwaZulu-Natal.^31^ Public facilities need to provide adequate resources to enable optometrists to fit and provide contact lenses to KC patients.

Recommendations arising from this study are that hospitals should invest in upskilling their optometrists through programmes to acquire up-to-date clinical knowledge, skills and competencies in KC patients’ care. Optometrists need to be able to perform the full spectrum of care for KC patients as defined by the regulatory authority minimum competency document.^50^ Upskilling programmes are usually mandated across healthcare professions for specific purposes such as when the scope of practice is increased or for special needs such as the case of KC.^51,52^ Optometrists in this study have shown an interest in enrolling in such programmes. They are mostly young adults and therefore are likely to embrace the challenges with their knowledge and skills of KC patient care given their improvement.

Hospitals need to develop protocols for screening, examination, diagnosis and management of all patients, including KC patients. The protocols should also define the level of service to be provided at each hospital and further define the type of resources including equipment required per hospital. In addition to the protocols, hospitals should further revise their policy level of service such that it adheres to the scope of practice of optometry. This should be accompanied by the provision of resources that enable practising a full scope for all professions. Optometrists in all hospitals should be able to fit contact lenses and manage contact lens–wearing patients, and in cases where the fitting is not possible, they should at least be able to support contact lens–wearing patients for their aftercare consultations.

Thirdly, hospitals need to invest in the provision of appropriate equipment that would enable optometrists to screen, examine and diagnose KC with ease at nascent stages. In addition, they must provide resources to enable optometrists to fit, dispense and provide aftercare of contact lenses to consulting KC patients. While equipment such as optical coherence tomographers (OCTs), Pentacams and pachymeters are necessary at the referral centres, all hospitals require basic equipment such as slit lamp biomicroscopes and keratometers to enable basic KC screening and examination and to conduct contact lens fitting and aftercare consultation sessions.

One of the limitations of this study is that self-reported knowledge and skills were evaluated. In addition, the study area was purposively selected and therefore determined a limited sample size. Future studies should consider the objective evaluation of the knowledge and skills of optometrists to ascertain their abilities to provide KC patient care. A broader study area, such as provincial or national context, may also be considered to include a larger sample size for better inferences of the findings.

## Conclusion

The appropriate diagnosis and management of KC are important to preserve and correct the vision of the KC patient so that they maintain a good quality of life. This study found that 45% of optometrists lacked adequate knowledge and skills for examination, diagnosis and management of KC patients, 79.2% of optometrists reported their respective hospitals did not have relevant equipment and only three (12.5%) of the 24 optometrists fitted contact lenses to manage KC patients. The study further indicated that only three of the seven hospitals had the equipment to conduct external examinations and evaluate the corneal curvature. The lack of equipment and resources were considered to contribute to and impede the ability of optometrists to diagnose and manage KC patients. Optometrists who were employed for longer periods in the public hospitals had a lack of knowledge and skills as compared with those more recently employed in this district. It is recommended that Capricorn district public hospitals should invest in regular upskilling for optometrists, develop relevant protocols for management of KC patients in line with the resources available and provide sufficient and appropriate equipment for diagnosis of KC and management with contact lenses.
